# Antibiogram Screening and Detection of Virulence-Associated Genes in *Brucella* Species Acquired from Cattle in South Africa’s Eastern Cape Province

**DOI:** 10.3390/ijerph19052813

**Published:** 2022-02-28

**Authors:** Rudzani P. Manafe, Nolwazi L. Bhembe-Magadaza, Ezekiel Green

**Affiliations:** Department of Biotechnology and Food Technology, University of Johannesburg, 55 Beit Street, Doornfontein, Johannesburg 2028, South Africa; rudzanimanafe@gmail.com (R.P.M.); egreen@uj.ac.za (E.G.)

**Keywords:** brucellosis, virulence associated, *Brucella*, multiple antibiotic resistance (MAR), *Brucella melitensis*, *Brucella abortus*, prevalence, putative, zoonotic

## Abstract

Brucellosis is a widespread zoonotic illness, and it poses serious public health and economic risks. The purpose of this investigation is to look at the antimicrobial susceptibility of unpasteurized milk, blood, and lymph node specimens from cattle, goats, and sheep, as well as to identify virulence-associated genes. In this investigation, a total of 123 isolates were examined. The activity of 15 antimicrobials against *Brucella* pathogens were assessed using the Kirby–Bauer disk diffusion technique. Nine virulence factors were detected with polymerase chain reaction analysis. Five antibiotics were 100% effective against *Brucella* isolates. A high level of resistance (100%) was documented with streptomycin, penicillin, and seven more antibiotics. Doxycycline resistance was found in 12% of goat isolates, and tetracycline resistance was found in 21% and 44% of goat and sheep isolates, respectively. Multiple antibiotic resistance (MAR) index >0.2 was found in 38.2% (47/123) of *Brucella* isolates. *VecC* and *BetB*, two *B. abortus* genes, were confirmed to be comparable. The findings of this study suggests that *Brucella* spp. are reservoirs of antibiotic resistance in the Eastern Cape Province. As such, they represent a potential pool of antibiotic genes that might be transferred to other pathogens in the community, and thus continue to pose a healthcare hazard.

## 1. Introduction

Brucellosis is a disease that is most commonly associated with domestic animals and marine creatures. Humans can contract the sickness by eating contaminated food or coming into contact with infected animals [1]. Every year, an estimated 500,000 new cases of brucellosis are recorded around the world, making it one of the most common zoonoses [2]. In South Africa, even if the exact occurrence is not known, brucellosis is still considered to be a priority zoonotic disease, with the last recorded incidence rate of >0.2 per 100,000 population, which was the result of a survey conducted in 1956 to 1959 [3], but the department of health indicated an increase rate of between <0.1 and 0.3 per 100,000 population yearly. The uncontrolled movement of cattle and also the shortage of vaccinations for susceptible animals has contributed to this increase in the incidence rate across the country [4]. Bovine brucellosis can be seen across all the nine provinces in South Africa, and is very concentrated around the Highveld regions and central areas [5]. Because of poor compliance with regard to the vaccination and testing of brucellosis, many South African livestock farmers currently are in danger of having livestock that are positive for brucellosis [3].

South Africa is one of the countries in Africa where brucellosis knowledge is still not widely disseminated [6]. Brucellosis is classed as a “controlled animal disease” in South Africa. There were 139 reported cases in 2018, which spiked to 423 cases in 2019 [7].

The treatment of brucellosis has always been a global problem, with various combinations of antibiotics undergoing experiments. Because brucellosis is an intracellular bacterial infection that affects host macrophage cells, treatment requires medications that can penetrate macrophages and function amid their cytoplasm [8,9]. These antimicrobial agents include tetracyclines, rifampicin, trimethoprim–sulfamethoxazole, and streptomycin [10,11]. These antimicrobial agents are more effective in treating brucellosis when used independently or as a combination. However, Brucella isolates are becoming resistant to the antibiotics recommended by the World Organization Health (WHO) [12], which could be due to the misuse of antibiotics. Antimicrobial resistance in *Brucella* has recently emerged in brucellosis-endemic areas around the world [13]. Quinolones, tetracyclines, beta-lactams, aminoglycosides, and imipenem are still overused non-therapeutically to treat various human illnesses in several regions of the world [14,15]. Antimicrobials are misused, resulting in the rise of multidrug-resistant microorganisms [16,17,18]. Antimicrobials used to stimulate growth or as a prophylactic in farm animals lead to the development of resistant strains, and play a crucial role in their dissemination throughout the food chain [19]. Antimicrobial resistance in zoonotic infections is also a concern, as this will impede illness treatment options in both healthcare and veterinary settings [20].

The genus *Brucella* is classified according to the type of species they primarily infect; *B. melitensis* colonizes goats and sheep, *B. abortus* colonizes cattle, *B. suis* colonizes pigs, *B. neotomae* infects desert woodrats, *B. ovis* infects rams, and *B. canis* infects dogs [12,21]. Other species have also been isolated from cetacean and pinniped species, such as *B. ceti* and *B. pinnipedialis*, respectively [22], while *B. microti* have been detected in voles [23], and *B. inopinata* in human breast implants [24]. The final member of the Brucella species has a much lower zoonotic potential than the first three traditionally large species; however, due to their high zoonotic potential, the first three *Brucella* species may cause negative effects, such as animal abortions, resulting in significant economic losses (nationally and internationally). This has led to the implementation of programs that aim to eradicate brucellosis in a variety of animals, more especially cattle and pigs [25].

All members of the genus *Brucella* strongly resemble each other according to their genetic and immunological evidence [26]. As a result, their pathogenicity is determined by the development of virulence factors, such as antigenic heterogeneity, exopolysaccharides, exotoxins, exoenzymes, fimbriae, flagella, and secretion mechanisms [27,28,29,30]. Even so, there is a level of dissimilarities in virulence, while the level of virulence differs in animals, for example, between humans and guineapigs [31]. Exotoxins, endotoxic lipopolysaccharides (LPSs), cytolysins, capsules, functioning flagella, fimbria, plasmids, and apoptosis inductors, as well as other bacterial virulence factors, such as exotoxins, endotoxic LPSs, and cytolysins, are all lacking in *Brucella* [32]. The lipopolysaccharides have two components: a type 43 secretion system and a cyclic β1,2-glucans (CβG) that has a three-fold function: masking *Brucella* from immune system identification, protection from the host, and the evasion of the host’s immune system [32].

The type IV (T4SS) secretion mechanism for the excretion of bacterial macromolecules and proteins in the microbial cell envelopes belongs to the multi-protein class of complete genomes [33]. There are 12 subunits that make up the stretching needle complex (*Vir*B2), the core outer membrane complex (*Vir*B7, *Vir*B9, and *Vir*B10), the connector stem (undoubtedly made up of *Vir*B5 or *Vir*B10 fragments), the inner membrane complex (*Vir*B3, *Vir*B4, *Vir*B6, *Vir*B8, and the *Vir*B10 N-terminus), and the *Vir*B10 N-terminus (consisting of *Vir*B4 and *Vir*B11). The only *Brucella* components that do not play a role in virulence are *Vir*B1, *Vir*B7, and *Vir*B12, though the others do [34].

Further new proteins have been discovered, including *Brucella* putative effectors (BPE), *Brucella*-secreted proteins (Bsps), and *Btp*A, which are among the *Vir*B-co-regulated effectors (*Vce*), such as *Vce*A and C, as well as *Brucella* putative effectors (BPE), *Brucella*-secreted proteins (Bsps), *Btp*A (*Brucella* TIR domain-containing proteins), and *Prp*A (proline racemase protein A), which triggers *IL-10* secretion and causes immune system non-responsiveness [35,36].

Brucellosis has immense effects on cattle, milk, and other dairy-related products, as well as human infections. Bovine brucellosis requires further investigation in order to minimize new infections. The genetic predominance analysis of virulence-related genes is therefore very significant, especially in South Africa and even in Africa as a whole, for the understanding and prevention of the disease. The purpose of this research was to find out how antibiotic susceptibility differed between *B. abortus* and *B. melitensis* isolates from cattle, sheep, and goats in the Eastern Cape Province of South Africa, in addition to the occurrences of nine putative genes linked to pathogenicity.

## 2. Materials and Methods

### 2.1. Ethics and Sample Collection

The University of Fort Hare Research Ethics Committee (UREC) approved the study, and the ethical clearance certificate REC-270710-028-RA Level 01 was issued. One thousand nine hundred and fifty-five samples (milk, blood, and lymph nodes) were acquired from several districts in the Eastern Cape Province. Briefly, the 1955 samples were random samples taken from cattle slaughtered in Queenstown and East London abattoirs, as well as 880 cattle, 555 sheep, and 520 goats from the livestock production sector of the Amathole District Municipality, Buffalo City Metropolitan Municipality, and OR Tambo District Municipality.

Cattle blood samples were taken from the caudal tail vein, whereas sheep and goat blood samples were taken from the jugular vein. Individual needles were used to capture all samples, which were then preserved in sterile EDTA vacutainer containers [37]. To avoid blood clotting, the tubes containing blood were tilted and placed on ice until additional investigation could be conducted. Raw milk samples were collected in individual sterile bottles from each quarter of dairy cows, sheep, and goats and stored at 4 °C for subsequent analysis [38]. Tissue samples of the mammary lymph nodes from calves slaughtered at Queenstown and East London abattoirs were collected and processed for bacterial isolation. Protocols for the collection of suspected material by district meat inspectors for the abattoir prevalence study were created after consultations with regional veterinary officers. Lymph nodes were randomly selected, and larger lymph nodes were also gathered using a straightforward purposive sampling method.

### 2.2. Bacterial Isolation

Milk samples were centrifuged for 15 min at 8753 r/min. After discarding the skimmed milk, the cream and deposit were combined and spread over Brucella agar (Merck, Johannesburg, South Africa), with Brucella supplement using a swab-stick (Liofilchem, Roseto D.A., Italy). The plates were incubated at 37 °C with a CO_2_ concentration of 5–10%. After 2, 4, and 7 days, the presence of Brucella colonies was checked. Blood from cattle, sheep, and goats was injected into a Castaneda biphasic media (Merck, Johannesburg, South Africa), which included both a solid and liquid Brucella medium, as well as a Brucella supplement (Liofilchem, Roseto D.A., Italy). The Castaneda bottles were incubated for 21 days with 5% CO_2_ supplementation and occasional flipping [37,39]. Lymph nodes were chopped into small pieces and dispersed on the surface of Brucella agar after being submerged in alcohol and flamed [38].

### 2.3. DNA Extraction and Confirmation Bru Gene (Brucella Species)

A Wizard Genomic DNA Purification kit (Promega ^®^ Corporation, Madison, WI, USA) was employed in the extraction of deoxyribonucleic acid (DNA) from 123 isolates, including 74 (60.2%) cattle, 33 (26.8%) goats, and 16 (13%) sheep, in accordance with the manufacturer’s recommendations. For the identification of *Brucella* sequences, genus-specific primers (Bru-F CTATTATCCGATTGGTGGTCTG and Bru-R GGTAAAGCGTCGCCAGAAGG) were employed [40]. A 1.5% agarose gel was prepare with 1 × TBE buffer (10 × TBE buffer: 1 M Tris, 1 M Boric acid, 50 mM EDTA, pH 8.3). Five microliters of 1 µg/mL ethidium bromide was used to stain the gel [41]. As size standards, a KAPA universal DNA molecular weight marker and a Fermentas 100 bp ladder were utilized. The amplicons were visualized under UV light and photographed using an Alliance 4.7 XD-79 System after gel electrophoresis at 100 V for 45 min (Uvitec, Cambridge, UK).

### 2.4. Antibiotic Susceptibility

#### 2.4.1. Antibiotic Susceptibility Testing

*Brucella* isolates’ susceptibility to 15 antibiotics (ciprofloxacin (5 µg), rifampicin (5 µg), amoxicillin (10 µg), doxycycline (30 µg), tetracycline (30 µg), trimethoprim and sulfamethoxazole (2.5 µg), ampicillin (10 µg), erythromycin (5 µg), ofloxacin (5 µg), cefixime (5 µg), moxifloxacin (5 µg), gentamicin (10 µg), penicillin G (10 units), levofloxacin (5 µg), and cefoxitin (30 µg)) was used to determine the results using the Kirby Bauer disk diffusion method [42]. Briefly, bacterial suspensions calibrated to a 0.5 McFarland standard turbidity were inoculated on Mueller–Hinton agar (Merck, Johannesburg, South Africa) plates supplemented with Brucella supplement (Liofilchem, Roseto D.A., Italy), and antibiotic disks (Mast Diagnostics, Merseyside, UK) were applied. Plates were then incubated for 48 h at 37 °C in 5% CO_2_, and zones of inhibition were classified as resistant or sensitive using the interpretative chart method, as per CLSI recommendations [43].

#### 2.4.2. Multiple Antibiotic Resistance

For the resistant isolates, multiple antibiotic resistance (MAR) traits, trends, and categorization were created [44]. The Krumperman formula was used to compute the MAR index of each of the detected isolates [45].

The MAR index of an isolate is the number of antibacterial agents to which it was resistant divided by the total number of antibacterial agents to which it was assigned. With an MRA grade of 0.2, antibiotics are regularly used in a high-risk context [46].

### 2.5. Molecular Detection of Putative Genes of Brucella

In Eastern Cape livestock, the genetic regularity of nine possible sources of virulence of two *Brucella* species was investigated. Oligonucleotide primers targeting the *Vir*B5 gene encoding the linking stalk of the T4SS, the *Vir*B2 gene for the stretching needle complex of the T4SS, the *Btp*A and *Btp*B genes for TIR proteins, the *Vce*C gene that is the *Vir*B-co regulator, the Bet gene coding for betaine aldehyde dehydrogenase, the BPE275 gene, the BSPB gene, and the *Prp*A virulence gene were amplified using PCR (Table 1), and a total of 25 µL reaction was used for the PCR assays [47]. Amplicons were run on 1.5% Agarose gel that was prepared using 1 × TBE buffer (10 × TBE buffer: 1 M Tris, 1 M Boric acid, 50 mM EDTA, pH 8.3) and stained with 5 µL of 1 µg/mL ethidium bromide. A Quick Load 1 kb DNA ladder was employed. The amplicons were seen under UV light and photographed using a UV transilluminator (UVP Chem doc, Bio-Rad^®^, Hercules, CA, USA) after 45 min of gel electrophoresis at 100 Volts.

## 3. Results

### 3.1. Bacterial Isolate Confirmation Using Polymerase Chain Reaction

The *Bru* gene was effectively amplified from 123 (6.3%) of the 1955 samples analyzed. An agarose gel confirmed that the targeted gene had been amplified and had a base pair size of 245 on it. Cow samples had the most isolates, with 74 (60.2%), followed by goat samples with 33 (26.8%), and sheep samples with 16 (13%).

### 3.2. Antibiogram Profile

In this study, *Brucella* isolates were 100% susceptible to moxifloxacin, gentamicin, levofloxacin, ofloxacin, and cefixime. These antibiotics may be used in the management of brucellosis in cattle, goats, and sheep. Intermediate resistance was observed with doxycycline (12%) in goats isolates, and tetracycline at 21% and 44% for both goats and sheep, respectively (Table 2).

### 3.3. The Phenotype of Multiple Antibiotic Resistance (MAR) and MAR Indices (MARI)

Table 3 shows the phenotyping of *Brucella* spp. for their MAR phenotypes and MAR indices (MARI). At least five drugs were resistant in 47 isolates. A total of five to eleven antibiotics were found to have multiple antibiotic resistances. The most common MAR phenotype was A5: CIP^R^ E^R^ PG^R^ RP^R^ A^R^ DXT^R^ T^R^ SXT^R^ AP^R^, which occurred in 35% (43/123) of the *Brucella* isolates. The MARI for all of the isolates, on the other hand, ranged from 0.3 to 0.7, with the mean being 0.5.

### 3.4. Frequency of Putative Genotypes in B. melitensis and B. abortus Isolates

The complete occurrence of *bet*B and *bsp*B were observed in 73% and 70.8%, respectively, in the two strains investigated. The highest occurrence of *bet*B, *vce*C, and *bsp*B (100%) was observed from *B. abortus,* while the lowest occurrence was observed in *btp*A (7%) and *prp*A (5.6%). There was no *vir*B2 and *prp*A detected (0%) in *B. melitensis*, while *bet*B (34.7%) was the highest virulent determinant observed for *B. melitensis*. Only 120 isolates out of a total of 123 isolates had the targeted virulence genes (Table 4).

## 4. Discussion

Brucellosis is spread through the mucosa, incised epidermis, secretions, and debris from an aborted fetus or an infected animal body [47]. These infections are employed to infiltrate host cells in a stealthy manner in order to prevent bactericidal reactions in macrophages [21,48,49,50,51,52,53,54]. To our knowledge, there is very little information on the molecular characterization of *Brucella* spp. pathogenic genes isolated from South African cattle, sheep, and goats. The pathogenicity of *Brucella*’s cell envelope protein [54], as well as the functioning of the genes associated with this envelope protein, must adjust to environmental stress, intracellular modulatory activity, and the ability to survive [55]. The first connection between the two usually determines pathogen colonization or eradication in the host cell, because it begins signals that cause changes in the gene expression features in the cell envelope proteins [52,56,57].

Livestock may be an effective medium for the propagation of antimicrobial-resistant *Brucella* spp. in the population. It was therefore hypothesized that animals might become reservoirs of infections contributing to the spread in populations of pathogenic bacteria, which are particular of multidrug resistant forms [58]. Studies on drug-resistant *Brucella* isolates in South Africa are also essential. Antibiotic resistance was found to be 100% for streptomycin, penicillin G, erythromycin, ampicillin, amoxicillin, trimethoprim–sulfamethoxazole, and rifampicin in this investigation, which is comparable to what has been found in other studies [49,59]. Another study found 100% susceptibility to penicillin G and erythromycin, which contradicts the findings of this study [60]. For brucellosis treatment, trimethoprim–sulfamethoxazole (TS) is highly recommended, especially in children under the age of eight [46]. The resistance of *Brucella* to β-lactam antibiotics can be related to the popular use of penicillin in the treatment of animal diseases, which results in an increase in resistance to the antibiotics of β-lactam. *Brucella* isolates were susceptible to moxifloxacin, gentamicin, levofloxacin, ofloxacin, and cefixime (100%). These antibiotics may be used in the management of brucellosis in cattle, goats, and sheep. Intermediate resistance was observed with doxycycline (12%) in goat isolates and tetracycline at 21% and 44% for both goats and sheep, respectively. According to a study, organisms with intermediate resistance are more likely to become resistant [61]. Multidrug resistance was found in 39.1% of the isolates, with diverse forms of multiple antibiotic resistance phenotypes (MARP). The CIP^R^ E^R^ PG^R^ RP^R^ A^R^ DXT^R^ T^R^ SXT^R^ AP^R^ CFM^R^ FOX^R^ was the most common MARP, detected in 11 (9.1%) isolates from various pathotypes. Other MARP with resistance to five to nine different antibiotics were also found at various frequencies. One of the biggest effects of multiple antibiotic resistance is the restricted number of effective treatments available to combat brucellosis, which was previously considered to be curable. The reclassification of many illnesses as recurrent with relevant clinical effects, such as prolongation of the illness, higher therapy costs, and an elevated risk of mortality, has been influenced by multidrug resistance. The multiple antibiotic resistance index (MARI) was used to measure the health hazards associated with the spread of pharmacological resistance in the community.

A multiple antibiotic resistant index (MARI) value of 0.2 (arbitrary) is used to distinguish between low and high infection risk, and a MARI value greater than 0.2 indicates that a bacteria strain was obtained from a high-pollution environment or from heavy antibiotic use [46]. A significant number of Brucella isolates exhibited a multiple antibiotic resistance (MAR) index of greater than 0.2, indicating that they came from high-risk sources and had been exposed to antibiotics previously. The MARI estimates for isolates from this investigation ranged from 0.3 to 0.7, and were more than 0.2, indicating that the isolates came from areas where antibiotics were widely used or contaminated. The high MARI values found in this study could indicate that the isolates were exposed to antibiotic pressure, which could have been as a consequence of unsuitable antibiotic usage within the populace in the studied region, and could result in an increment in multidrug resistance emergence over time if appropriate measures are not taken.

When all 120 samples were analyzed for the nine virulence genes, it was discovered that a significant number of these genes were expressed in *B. abortus* samples, with *B. melitensis* accounting for only a tiny number of genes expressed. *Brucella melitensis* has been the reported to be the more virulent spp. [62]. The *B. abortus* strain has shown to be more isolated than *B. melitensis* in this investigation. The results of screening the nine putative virulence genes of *B. melitensis* and *B. abortus* from our samples revealed the presence of *Vir*B5, *Btp*A, *Btp*B, *Vce*C, *Bet*B, *BPE*275, *Vir*B2, *BSPB*, and *Prp*A in all isolated isolates in the current investigation. However, *Vir*B2 (91.5%) and *Prp*A (5.6%) were found in only *B. abortus* samples. Despite lacking some of the typical characteristics of virulence and strength, such as capsules, fimbria, plasmids, exotoxins, lysogenic phages, and endotoxic lipopolysaccharide, Brucellae did express certain pathogenic genes.

The *Bet*B, *BSPB*, and *Vec*C genes were found in 100% of the *B. melitensis* isolates, according to our data. One of the most common virulence genes in this study is *Bet*B, which encodes betaine dehydrogenase (BADH). This gene converts betaine aldehyde to glycine betaine, which enables all eukaryotic and prokaryotic cells to maintain osmotic stress stability [62]. The *Bsp*B gene inhibits or stops the protein secretory pathway of the host-activated cells that are infected, and also they are responsible for preventing cellular secretion during the infection of cells [35]. The existence of this gene is alarming, as this indicates that a great deal is still to be understood about the *Brucella* species. *Vce*C is a highly conserved type IV secretion system (T4SS) effector, with major effects on autophagy and apoptosis seen in all sequenced Brucella genomes [63,64]. In this study, *Vir*B5 and *Vir*B2, which are required for intracellular survival, were found in 6.1% of *B. melitensis* and 14.1% and 91.5% of *B. abortus* samples, respectively. *Vir*B is one of the most essential virulence factors. The *Vir*B gene encodes one of the primary virulence features required for the bacterium’s intracellular lifestyle, and is used for virulence factor translocation into the host cell. Another study reported 73.8% of *Vir*B among 42 *B. melitensis* strains isolated from aborted fetuses of sheep and goats [65]. Furthermore, *Btp*A and *Btp*B were also detected from the *B. melitensis* and *B. abortus* isolates. The proteins *Btp*A and *Btp*B have been shown to be translocated by *Brucella* into host cells [66]. In vitro and overexpression investigations have demonstrated that *Btp*A firmly binds with microtubules and is able to stabilize polymerized microtubules by blocking microtubule disassembly triggered by the microtubule drug nocodazole [67]. It has been demonstrated that *Btp*B, like *Btp*A, can protect microtubules from drug-induced depolymerization [68]. This study also reports the *BPE*275 and the *Prp*A gene that were also detected. This is in accordance with a study of 60 *B. melitensis* isolates from Iran that reported on them [69].

There are genes associated with virulence or organism survival; research into these genes will aid in the development of safe and protective vaccines, such as live but attenuated vaccines, which provide significant amounts of protection [70]. Purine biosynthesis pathway genes ferro chelatase hem H mutant, a gene required to transport lipid A 363 fatty acid, the phosphoglycerate kinase-encoding gene, LPS biosynthesis pathway genes, and 364 Type IV secretion *Vir*B genes are just a few examples of vaccines developed using deletions in *B. abortus* virulence genes that result in major attenuation [71,72,73,74,75,76].

## 5. Conclusions

The findings of this investigation specifically demonstrates that cattle, goats, and sheep in the Eastern Cape are reservoirs of *Brucella* spp. that are resistant to antibiotics, and that they have a potential pool of antibiotic-resistant genes that may be spread to other pathogens in the population, posing a risk to public health. This situation is worrisome, as certain severe bacterial infections may lack therapeutic options in the near future. This problem, together with a large number of immunocompromised citizens in South Africa, demands that the spread of antibiotic resistance be monitored as a priority to ensure the health of the general population. The different virulence factors responsible for its pathogenicity shows that infection to a host cell transpires in stages. Cattle remains the major carrier of *Brucella* spp., with *B. abortus* the primarily strain isolated. Because vaccines are not readily available, especially in disadvantaged communities, there is still a need to educate the general public about this disease; therefore, public health education should focus on the zoonotic features of the disease as it pertains to the consumption of unpasteurized milk and other food stuffs sourced from infected animals.

## Figures and Tables

**Table 1:** List of primers and PCR conditions used for the amplification of *Brucella* virulence-associated genes.

Gene	Primer Sequence (5′-3′)	PCR Conditions	Amplicon Size (bp)	Reference
*Vir*B5	VirB5-F: ATTCTCAGCTTCGCATTC VirB5-R: TCACCGCTTCGTAGAGAT	Denaturation at 94 °C for 4 min, then 30 cycles of heat denaturation at 94 °C for 60 s, primer annealing at 56 °C for 45 s, and DNA extension at 72 °C for 1 min. To complete the synthesis of all strands, a last extension at 72 °C for 10 min is required.	274	[47]
*Btp*A	BtpA-F: CTATCAGGCTAAGCAATTC BtpA-R: CGTAGGAAACTTTATGCC	Denaturation at 94 °C for 4 min, then 30 cycles of heat denaturation at 94 °C for 60 s, primer annealing at 56 °C for 45 s, and DNA extension at 72 °C for 1 min. To complete the synthesis of all strands, a last extension at 72 °C for 10 min is required.	458	[47]
*Btp*B	BtpB-F: TTAACCAGCACGAATACACG BtpB-R: CTACGATCAGTTTGCAGCG	Denaturation at 94 °C for 4 min, then 30 cycles of heat denaturation at 94 °C for 60 s, primer annealing at 61 °C for 45 s, and DNA extension at 72 °C for 1 min. To complete the synthesis of all strands, a last extension at 72 °C for 10 min is required.	579	[47]
*Vce*C	VceC-F: CGCAAGCTGGTTCTGATC VceC-R: TGTGACGGGTAATTTGAAGC	Denaturation at 94 °C for 4 min, then 30 cycles of heat denaturation at 94 °C for 60 s, primer annealing at 61 °C for 45 s, and DNA extension at 72 °C for 1 min. To complete the synthesis of all strands, a last extension at 72 °C for 10 min is required.	482	[47]
*Bet*B	BetB-F: GCTCGAAACGCTGGATAC BetB-R: AGGCGATGATTGACGAGC	Denaturation at 94 °C for 4 min, then 30 cycles of heat denaturation at 94 °C for 60 s, primer annealing at 60 °C for 45 s, and DNA extension at 72 °C for 1 min. To complete the synthesis of all strands, a last extension at 72 °C for 10 min is required.	393	[47]
*BPE275*	BPE275-F: TGTCGCGGTCTATGTCTATC BPE275-R: AATGAGGACGGGCTTGAG	Denaturation at 94 °C for 4 min, then 30 cycles of heat denaturation at 94 °C for 60 s, primer annealing at 59 °C for 45 s, and DNA extension at 72 °C for 1 min. To complete the synthesis of all strands, a last extension at 72 °C for 10 min is required.	466	[47]
*Vir*B2	VirB2-F: GCTGTCGCGGATTCTACC VirB2-R: CGGAATGCCATCTTGTAAC	Denaturation at 94 °C for 4 min, then 30 cycles of heat denaturation at 94 °C for 60 s, primer annealing at 60 °C for 45 s, and DNA extension at 72 °C for 1 min. To complete the synthesis of all strands, a last extension at 72 °C for 10 min is required.	198	[47]
*BSP*B	BSPB-F: TATCCATGGTATATGCGCC BSPB-R: ATAAAGGCCGGGAATGAC	Denaturation at 94 °C for 4 min, then 30 cycles of heat denaturation at 94 °C for 60 s, primer annealing at 62 °C for 45 s, and DNA extension at 72 °C for 1 min. To complete the synthesis of all strands, a last extension at 72 °C for 10 min is required.	336	[47]
*Prp*A	PrpA-F: AACCTCAATGGATCGACC PrpA-R: ACGGTCGATAGCCTTGTC	Denaturation at 94 °C for 4 min, then 30 cycles of heat denaturation at 94 °C for 60 s, primer annealing at 58 °C for 45 s, and DNA extension at 72 °C for 1 min. To complete the synthesis of all strands, a last extension at 72 °C for 10 min is required.	672	[47]

**Table 2:** Antibiotic susceptibility testing of *Brucella* isolated from cattle, goats and sheep.

Antibiotics	Cattle			Total (%)			Goats			Total (%)			Sheep			Total (%)		
	S	I	R	S	I	R	S	I	R	S	I	R	S	I	R	S	I	R
Ciprofloxacin (10 µg)	9	0	65	12	0	88	33	0	0	100	0	0	16	0	0	100	0	0
Rifampicin (5 µg)	0	0	74	0	0	100	0	0	33	0	0	100	0	0	16	0	0	100
Amoxicillin (10 µg)	0	0	74	0	0	100	0	0	33	0	0	100	0	0	16	0	0	100
Doxycycline (30 µg)	0	0	74	0	0	100	0	4	29	0	12	88	0	0	16	0	0	100
Tetracycline (5 µg)	0	0	74	0	0	100	0	7	26	0	21	79	0	7	9	0	44	56
Trimethoprim–sulfamethoxazole (2.5 µg)	0	0	74	0	0	100	33	0	0	100	0	0	16	0	0	100	0	0
Ampicillin (10 µg)	0	0	74	0	0	100	0	0	33	0	0	100	0	0	16	0	0	100
Erythromycin (15 µg)	0	0	74	0	0	100	0	0	33	0	0	100	0	0	16	0	0	100
Ofloxacin (5 µg)	74	0	0	100	0	0	33	0	0	100	0	0	16	0	0	100	0	0
Cefixime (5 µg)	61	0	13	82	0	18	33	0	0	100	0	0	16	0	0	100	0	0
Moxifloxacin (5 µg)	74	0	0	100	0	0	33	0	0	100	0	0	16	0	0	100	0	0
Gentamicin (10 µg)	74	0	0	100	0	0	33	0	0	100	0	0	16	0	0	100	0	0
Penicillin G (10 units)	0	0	74	0	0	100	0	0	33	0	0	100	0	0	16	0	0	100
Levofloxacin (5 µg)	74	0	0	100	0	0	33	0	0	0	0	100	16	0	0	100	0	0
Cefoxitin (30 µg)	0	0	74	0	0	100	33	0	0	100	0	0	16	0	0	100	0	0

Susceptibility (S), Intermediate (I), Resistance (R).

**Table 3:** Antibiotypes and MARI of *Brucella* isolates.

Antibiotic Code	Antibiotype	Number of Antibiotics	MARI
A1	E^R^ PG^R^ RP^R^ A^R^ AP^R^	5	0.3
A2	E^R^ PG^R^ RP^R^ A^R^ DXT^R^ AP^R^	6	0.5
A3	E^R^ PG^R^ RP^R^ A^R^ DXT^R^ T^R^ AP^R^	7	0.5
A4	E^R^ PG^R^ RP^R^ A^R^ DXT^R^ T^R^ SXT^R^ AP^R^ FOX^R^	9	0.6
A5	CIP^R^ E^R^ PG^R^ RP^R^ A^R^ DXT^R^ T^R^ TS^R^ AP^R^	9	0.6
A6	CIP^R^ E^R^ PG^R^ RP^R^ A^R^ DXT^R^ T^R^ SXT^R^ AP^R^ CFM^R^ FOX^R^	11	0.7

Amoxicillin (A), ciprofloxacin (CIP), doxycycline (DXT), penicillin G (PG), cefixime (CFM), trimethoprim/sulfamethoxazole (SXT), rifampicin (RIF), erythromycin (E), cefoxitin (Fox), tetracycline (T), ampicillin (AP).

**Table 4:** Prevalence of genes linked to pathogenicity in the 120 Brucella isolates from livestock.

Target Strains	Number (%)	Number of Putative Virulence Genes in Studied Strains								
		*Vir*B5	*Btp*A	*Btp*B	*Vce*C	*Bet*B	*BPE*275	*Vir*B2	*BSP*B	*Prp*A
*B. melitensis*	49 (40.8)	3 (6.1)	2 (4.1)	1 (2)	9 (18.4)	17 (34.7)	3 (6.1)	0	14 (28.6)	0
*B. abortus*	71 (59.2)	10 (14.1)	24 (33.8)	5 (7)	71 (100)	71 (100)	70 (98.6)	65 (91.5)	71 (100)	4 (5.6)
*TOTAL*	120 (100)	13 (11)	26 (22)	6 (5)	80 (67)	88 (73)	73 (61%)	65 (54)	85 (70.8)	4 (3)

## Data Availability

Data is available from the authors at the University of Johannesburg if required.
